# Infliximab treatment reduces depressive symptoms in patients with ankylosing spondylitis: an ancillary study to a randomized controlled trial (ASSERT)

**DOI:** 10.1186/s13075-020-02305-w

**Published:** 2020-09-29

**Authors:** Casper Webers, Carmen Stolwijk, Olga Schiepers, Thea Schoonbrood, Astrid van Tubergen, Robert Landewé, Désirée van der Heijde, Annelies Boonen

**Affiliations:** 1 Department of Internal Medicine, Division of Rheumatology, Maastricht University Medical Centre, PO Box 5800, Maastricht, 6202 AZ The Netherlands; 2 Care and Public Health Research Institute (CAPHRI), Maastricht University, Maastricht, the Netherlands; 3 Department of Internal Medicine, Erasmus Medical Centre, Rotterdam, the Netherlands; 4 Department of Psychiatry and Neuropsychology, School for Mental Health and Neuroscience (MHeNs), Maastricht University, Maastricht, the Netherlands; 5 Department of Clinical Immunology & Rheumatology, Amsterdam Rheumatology Centre, University of Amsterdam, Amsterdam, the Netherlands; 6 Department of Rheumatology, Zuyderland Medical Centre, Heerlen, the Netherlands; 7 Department of Rheumatology, Leiden University Medical Centre, Leiden, the Netherlands

**Keywords:** Ankylosing spondylitis, Depressive symptoms, Anti-TNF-α therapy, Randomized controlled trial

## Abstract

**Background:**

Patients with ankylosing spondylitis (AS) are at increased risk of depression. This increased risk has been hypothesized to be solely secondary due to AS-related symptoms, or additionally due to a common inflammatory pathway. From a clinical perspective, it is important to know whether treatment with tumor necrosis factor alpha inhibitors reduces depressive symptoms, while from a pathophysiological point of view, it would be insightful to understand whether such an effect would be a direct result of reduced inflammation, the result of reduced AS-related symptoms, or both. The objective of this study was to evaluate the effect of infliximab on depressive symptoms in patients with AS in a randomized-controlled trial setting.

**Methods:**

Data were retrieved from a subgroup of patients from the AS Study for the Evaluation of Recombinant Infliximab Therapy (ASSERT). Patients were randomly allocated to infliximab (*n* = 16) or placebo (*n* = 7) until week 24, after which all received infliximab until week 54. Associations between treatment group and depressive symptoms, measured with the Center for Epidemiological Studies Depression scale (CES-D, range 0–60 (best-worst)) at baseline and over time, were explored with generalized estimating equations (GEE).

**Results:**

Mean CES-D score at baseline was 15.5 (SD 9.3) in the infliximab group and 17.3 (SD 5.7) in the placebo group. Twelve patients (52%) had a CES-D score > 16, suggestive for clinical depression. After 24 weeks, mean CES-D had decreased to 9.5 (SD 11.4) in the infliximab group, but was 18.0 (SD 6.9) in the placebo group. GEE revealed larger improvements in depressive symptoms (*B* = − 6.63, 95%CI − 13.35 to 0.09) and odds of possible depression (OR = 0.02, 95%CI 0.00 to 0.72) in the infliximab group, compared to the placebo group. Both associations largely disappeared when adjusted for self-reported disease activity and/or physical function. Additional adjustment for C-reactive protein (CRP) did not change results.

**Conclusions:**

Depressive symptoms are common in patients with AS and active disease. Infliximab improves these depressive symptoms in AS when compared to placebo by improving disease symptoms. We did not find an indication for a direct link between CRP-mediated inflammation and depressive symptoms.

**Trial registration:**

Trial registration (ASSERT): NCT00207701. Registered on September 21, 2005 (retrospectively registered).

## Background

Ankylosing spondylitis (AS) affects not only the patients’ physical health, but also their mental well-being. A systematic review showed that patients with AS have an increased prevalence of depressive symptoms, with rates ranging between 11 and 64% [1]. Comorbid depression in AS impacts the individual and has societal relevance, as it has been associated with work disability in inflammatory arthritis [2].

Different possible pathways might explain the increased prevalence of depressive symptoms in AS. Depressive symptoms could be secondary to disease-related impairments such as pain and limitations in physical functioning, or to psychological consequences such as worrying about the future [3]. But AS and depressive symptoms may also share a common pathophysiological pathway as both may be the result of an auto-inflammatory biological process; pro-inflammatory biomarkers, such as C-reactive protein (CRP) and tumor necrosis factor (TNF)-α, have been found to be higher in depressed individuals compared to non-depressed individuals, leading to the “inflammatory/cytokine hypothesis of depression” [4]. Also, a randomized controlled trial revealed that TNF-α inhibitors (TNFi) can improve depressive symptoms in patients with treatment-resistant depression and increased inflammatory markers [5].

From a clinical perspective, it is of importance to understand whether treatment with TNFi can reduce depressive symptoms in patients with AS. From a pathophysiological point of view, it might be interesting to understand if such an effect would be the result of a reduction of AS-related symptoms, inflammation, or both. Previously, several studies have reported improvements in depressive symptoms in patients with AS after treatment with infliximab [6–8]. Interestingly, observations on the mechanism behind this (potential) effect of infliximab were not equivocal, as correlations of depressive symptoms with either AS-related symptoms (self-reported disease activity) [8] or inflammatory biomarkers [6] have been reported. None of these studies was placebo-controlled or blinded, however, and the observed improvements could have been the result of regression to the mean. In two randomized controlled trials (RCTs), etanercept improved depressive symptoms in patients with AS and non-radiographic axial spondyloarthritis (nr-axSpA), although reported effects were small, and the nature of the effect of TNFi on depressive symptoms was not further explored [9, 10].

The objectives of the present study were to evaluate in patients with AS the effect of infliximab on depressive symptoms compared to placebo and to explore the association between depressive symptoms and AS-related symptoms (experienced disease activity and physical functioning) and inflammation over time.

## Patients and methods

Data were retrieved from a sub-study of the Ankylosing Spondylitis Study for the Evaluation of Recombinant Infliximab Therapy (ASSERT), which originally randomized 279 patients. Patients from ASSERT that had been included in the Maastricht University Medical Centre were considered eligible for the current sub-study, and asked to complete a questionnaire on depressive symptoms in parallel to the protocol-required assessments of ASSERT. The ancillary study population consisted of 23 patients, 16 patients randomly assigned to infliximab and 7 to placebo.

### Study design

The design of the ASSERT RCT has been previously reported [11]. Briefly, patients were included in ASSERT if they were 18 years or older and classified as AS according to the modified New York criteria. Patients had to have a Bath AS Disease activity index (BASDAI) score > 4 and a spinal pain assessment score > 4 on a visual analog scale [12]. Patients were randomly assigned (3:8 ratio) to receive infusions of 5 mg/kg infliximab or placebo at weeks 0, 2, 6, 12, and 18. From week 24 until week 54, all patients received infliximab therapy. The study protocol was reviewed and approved by the independent ethics committee (METC azM/UM). All patients provided written informed consent.

### Study outcomes

Depressive symptoms were assessed with the Center for Epidemiologic Studies Depression scale (CES-D) [13, 14]. This validated instrument was chosen since it contains fewer somatic items than other instruments [15]. The CES-D consists of 20 items on perceived mood and level of functioning during the past week. Every item is scored on a 4-point scale, where 0 = rarely or none of the time, 1 = some or little of the time, 2 = occasionally or a moderate amount of time, and 3 = most of the time. The total CES-D score is the sum of all items (range 0 [best] to 60 [worst]). In addition, four CES-D subscales have been defined (“Somatic-retarded activity” [range 0–21], “Depressed affect” [range 0–15], “Positive affect” [range 0–12] and “Interpersonal affect” [range 0–6]), reflecting combinations of varying individual items [16]. A total CES-D score of > 16 is employed as a cutoff suggestive for clinical depression (i.e., “possible depression”) and would warrant a referral for a diagnostic evaluation [14]. Self-reported disease activity and physical function were measured with the BASDAI and Bath AS Functional Index (BASFI), respectively [17]. Inflammation was assessed with the serum CRP. Study outcomes were assessed at weeks 0, 6, 12, 24, and 54. Both patients and assessors were blinded until week 24. The main interest of the study was the (between-group difference in) change from baseline CES-D at week 24, in line with ASSERT, in which the main outcome was assessed at the same point in follow-up.

### Statistical analysis

Differences in baseline characteristics between groups were explored with independent *t* test, Mann Whitney test, or chi-square test, depending on level of measurement and distribution. Fisher’s exact test was preferred over chi-square test for small samples (expected count < 5). Chi-square tests (or Fisher’s exact tests) and Mann-Whitney *U* tests were used to respectively compare the proportion of patients with a CES-D score > 16 and the mean CES-D scores between groups at the different time points.

The course of CES-D scores between groups over time (until week 24, as thereafter both groups were on infliximab) was compared using generalized estimating equation (GEE) analyses. GEE can take into account the within-subject correlation in a longitudinal study, i.e. the dependency that exists between assessments within the same subject [18, 19]. GEE is focused on estimating the average outcome in the population (population-averaged model), and the model estimates reflect both within-subject and between-subject effects. It requires an a priori defined “working correlation structure”. For this analysis, an “exchangeable” correlation structure was chosen, based on the similar correlations of CES-D scores between time points [19].

First, separate GEE analyses were carried out with either continuous CES-D scores, or dichotomized CES-D score (normal [< 16] vs. increased [≥ 16]), as the outcome (dependent variable). Considering the small sample size, only a limited number of variables could be included in the GEE. Group (infliximab versus placebo) and time (categorical) were included as independent variables, as was as an interaction between group and time (group*time), to test whether there was a difference in the outcome (change from baseline CES-D [continuous] or change from baseline odds of increased CES-D [dichotomized]) between the groups after the first 24 weeks. The GEE analyses were adjusted for baseline CES-D. Second, as we were specifically interested in the mechanism behind (improvement of) depressive symptoms in AS, we explored whether a potential association between infliximab and reduced depressive symptoms remained after adjustment for time-varying AS-specific variables of disease symptoms (BASDAI or BASFI; added as independent variables to the initial model with group, time, group*time and baseline CES-D as independent variables), as well as inflammation (CRP; added as independent variable to the initial model with group, time, group*time and baseline CES-D as independent variables), or both. Of note, BASDAI and BASFI were added in separate models due to collinearity. In ASSERT, treatment allocation was stratified by CRP (within or above 3 times the upper limit of normal); for the current sub-study, we did not adjust for this stratification, as CRP was one of our variables of interest and in light of the sample size. For all analyses, *p* < 0.05 was considered statistically significant. Analyses were performed with R, version 3.5.3 [20].

## Results

At baseline, the groups were largely comparable in terms of demographics and disease characteristics, although the placebo group contained only male patients and had slightly (though statistically non-significant) higher mean BASFI and Patient Global (Table 1). At weeks 6, 12, 24 and 54, a CES-D score was missing for 1, 2, 0 and 2 patients, respectively; none of the patients had more than 1 missing CES-D score. The mean CES-D score at baseline was 15.5 (SD 9.3) in the infliximab group and 17.3 (SD 5.7) in the placebo group. Fifty-six percent (9 of 16) of the patients in the infliximab group and 43% (3 of 7) in the placebo-group had a CES-D score > 16 at baseline, suggestive for clinical depression. The scores on the four CES-D subscales did not differ between groups at baseline (see Additional file 1). When comparing the current sub-study population (*n* = 23) to those who did participate in ASSERT but not part of this sub-study (*n* = 256), the ancillary study patients had slightly higher scores on BASDAI (7.0 [SD 1.1] versus 6.4 [SD 1.6]) and BASFI (6.6 [SD 1.5] versus 5.7 [SD2.0]) (see Additional file 2).

**Table 1:** Baseline characteristics separately for patients in the infliximab and placebo arm

	Infliximab (***n*** = 16)	Placebo (***n*** = 7)	*p*
Male gender, *n* (%)	11 (68.8)	7 (100.0)	0.27
Age, years	38.6 (11.6)	44.9 (5.8)	0.19
Disease duration, years	8.3 (8.2)	11.5 (7.4)	0.37
HLA-B27 positive, *n* (%)	14 (87.5)	5 (71.4)	0.56
History of uveitis, *n* (%)	6 (37.5)	1 (14.3)	0.37
History of psoriasis, *n* (%)	0 (0.0)	0 (0.0)	–
History of IBD, *n* (%)	2 (12.5)	1 (14.3)	1.00
BASDAI score, 0–10	7.0 (1.3)	7.1 (0.7)	0.55
BASFI score, 0–10	6.3 (1.5)	7.2 (1.3)	0.21
Patient’s global assessment, 0–10 VAS	6.8 (1.4)	7.8 (1.2)	0.15
Mander enthesis index, 0–90	7.7 (8.6)	12.8 (14.6)	0.69
Swollen joint index, 0–44	2.9 (3.7)	3.0 (3.9)	0.86
Chest expansion, cm	2.0 (0.9)	2.5 (2.0)	0.87
Night pain, 0–10 VAS	6.4 (2.0)	7.4 (1.0)	0.11
CRP level, mg/L	26.0 (24.4)	15.4 (16.0)	0.22
Increased CRP, *n* (%)*	13 (81.3)	6 (85.7)	1.00
CES-D score, 0–60	15.5 (9.3)	17.3 (5.7)	0.66
Increased CES-D, *n* (%)^†^	9 (56.3)	3 (42.9)	0.67

Values expressed as mean (SD), unless otherwise indicated

*HLA-B27* human leucocyte antigen-B27, *IBD* inflammatory bowel disease, *BASDAI* Bath Ankylosing Spondylitis Disease Activity Index, *BASFI* Bath Ankylosing Spondylitis Functional Index, *VAS* visual analog scale, *CRP* C-reactive protein, *CES-D* Center for Epidemiologic Studies Depression Scale

*Defined as CRP > 5 mg/L

^†^Defined as a score ≥ 16, indicating possible depression

### Course of CES-D over time

**Fig. 1:**
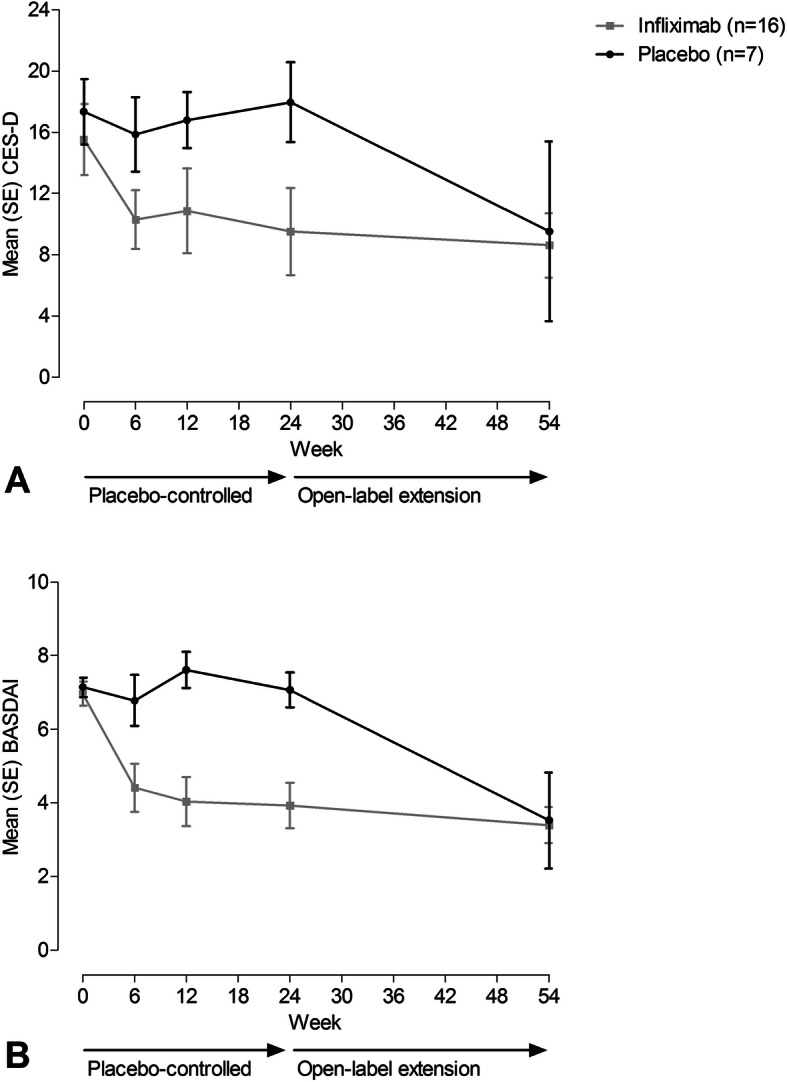
CES-D (**a**) and BASDAI (**b**) scores over time according to treatment group. Figure caption: Course of mean CES-D (**a**) and BASDAI (**b**) scores over time, by treatment group. During the open-label extension, all patients received infliximab. BASDAI, Bath Ankylosing Spondylitis Disease Activity Index; CES-D, Center for Epidemiologic Studies Depression Scale; SE, standard error

In the first 24 weeks after baseline, CES-D in the infliximab group decreased substantially compared to the placebo group (Fig. 1a). Within groups, the mean (SD) [change from baseline (Δ), number of patients who completed the CES-D at that time point] CES-D score at week 6 had decreased to 10.3 (SD 7.7) [Δ − 5.2, *n* = 16] in the infliximab group and 15.9 (SD 6.0) [Δ − 2.5, *n* = 6] in the placebo-group (*p* = 0.03 for comparison of absolute CES-D). At 24 weeks, mean CES-D scores were 9.5 (SD 11.4) [Δ − 6.0, *n* = 16] in the infliximab group and 18.0 (SD 6.9) [Δ + 0.6, *n* = 7] in the placebo group (*p* = 0.02 for comparison of absolute CES-D). At week 54, 30 weeks after the original placebo group had switched to infliximab, the mean CES-D score in the original placebo group had decreased to the same degree as the infliximab group (9.5 (SD 13.1) [Δ − 9.3, *n* = 5] vs. 8.6 (SD 8.4) [Δ − 6.9, *n* = 16], *p* = 0.90 for comparison of absolute CES-D). Similar changes over time were observed in both groups for BASDAI (Fig. 1b).

Exploration of the CES-D subscales showed scores in the infliximab group had improved strongest in the first 24 weeks after baseline for the subscale “Somatic-retarded activity” and to a lesser extent for the subscale “Depressed affect”, when compared to the placebo group (see Additional file 1).

**Fig. 2:**
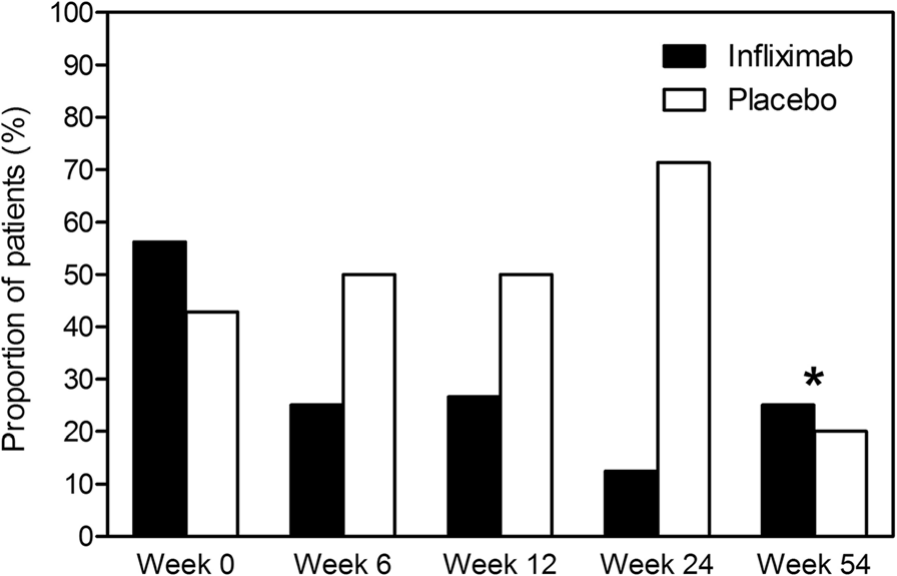
Proportion of patients with CES-D score ≥ 16 over time according to treatment group. Figure caption: Proportion of patients with an increased CES-D score (≥ 16) at each assessment, by treatment group. *From week 24 onwards, all patients received infliximab. CES-D, Center for Epidemiologic Studies Depression Scale

At week 6, 25% (4 of 16) of the infliximab group had a CES-D score > 16 suggestive for clinical depression, compared to 50% (3 of 6) in the placebo group (*p* = 0.33) (Fig. 2). After 24 weeks, these proportions were 13% (2 of 16) and 71% (5 of 7), respectively (*p* = 0.01). At week 54, when all patients received infliximab, 20–25% (1 of 5 of the original placebo group, 4 of 16 of the original infliximab group) had a CES-D score > 16.

### GEE analysis of factors associated with CES-D over time

Differences in disease course between treatment groups over time were explored by GEE. As expected, BASDAI scores in the first 24 weeks were significantly lower in the infliximab group compared to the placebo group (*B*_group*time(24)_ = − 2.97, 95%CI − 4.51 to − 1.42, *p* < 0.01). This effect was partially explained by a reduction in inflammation (reflected by CRP) in the infliximab group, as observed in separate models in which CRP was included in addition to treatment (data not shown).

Further exploration of depressive symptoms by GEE revealed a (borderline non-significant) larger improvement in CES-D scores in the infliximab group compared to the placebo group after the first 24 weeks (model 1A: *B*_group*time(24)_ = − 6.63, 95%CI − 13.35 to 0.09, *p* = 0.05). After adjustment for BASDAI, no association was observed anymore between infliximab and change in CES-D scores over time (model 1B: *B*_group*time(24)_ = − 2.66, 95%CI − 9.44 to 4.11, *p* = 0.44), while BASDAI itself was significantly associated with CES-D scores (model 1B: *B*_BASDAI_ = 1.34, 95%CI 0.53 to 2.15, *p* < 0.01) (Table 2). Adjustment for CRP instead of BASDAI had a notably smaller effect on the association between infliximab treatment and CES-D scores (model 1C: *B*_group*time(24)_ = − 5.71, 95%CI − 12.53 to 1.12, *p* = 0.10). Finally, when both BASDAI and CRP were included, BASDAI remained associated with CES-D scores, while CRP was not (model 1D, Table 2).

**Table 2:** Multivariable GEE analysis exploring the effect of infliximab on CES-D scores, BASDAI models

**Outcome = CES-D (absolute score)**	**Model 1A**			Model 1B*			Model 1C*			Model 1D*		
	***B***	**95% CI**	***p***	***B***	**95% CI**	***p***	***B***	**95% CI**	***p***	***B***	**95% CI**	***p***
Group, IFX	− 0.39	− 2.01 to 1.23	0.63	− 0.35	− 2.70 to 2.01	0.77	− 0.80	− 2.85 to 1.25	0.44	− 0.24	− 2.91 to 2.42	0.86
Baseline CES-D, score	0.78	0.46 to 1.11	< 0.01	0.68	0.45 to 0.91	< 0.01	0.77	0.43 to 1.11	< 0.01	0.68	0.45 to 0.92	< 0.01
Time, 6 weeks	− 2.30	− 8.09 to 3.49	0.44	− 1.39	− 5.77 to 2.98	0.53	− 2.31	− 8.14 to 3.53	0.44	− 1.37	− 5.75 to 3.00	0.54
Time, 12 weeks	− 0.42	− 5.63 to 4.78	0.87	− 1.16	− 6.26 to 3.95	0.66	− 0.58	− 5.88 to 4.72	0.83	− 1.13	− 6.27 to 4.00	0.67
Time, 24 weeks	0.62	− 4.69 to 5.92	0.82	0.71	− 4.43 to 5.86	0.79	0.42	− 4.92 to 5.75	0.88	0.77	− 4.48 to 6.02	0.77
Group*time (6 weeks)	− 2.94	− 9.23 to 3.35	0.36	− 0.43	− 5.71 to 4.85	0.87	− 2.24	− 8.92 to 4.44	0.51	− 0.57	− 6.02 to 4.89	0.84
Group*time (12 weeks)	− 4.22	− 10.32 to 1.87	0.17	0.44	− 6.23 to 7.11	0.90	− 3.27	− 9.69 to 3.16	0.32	0.27	− 6.44 to 6.99	0.94
Group*time (24 weeks)	− 6.63	− 13.35 to 0.09	0.05	− 2.66	− 9.44 to 4.11	0.44	− 5.71	− 12.53 to 1.12	0.10	− 2.83	− 9.68 to 4.01	0.42
BASDAI	−	−	−	1.34	0.53 to 2.15	< 0.01	−	−	−	1.36	0.50 to 2.23	< 0.01
CRP, mg/L	−	−	−	−	−	−	0.04	− 0.02 to 0.10	0.24	− 0.01	− 0.07 to 0.05	0.76
**Outcome = CES-D (increased vs. normal)**^**†**^	**Model 2A**			Model 2B*			Model 2C*			Model 2D*		
	**OR**	**95% CI**	***p***	**OR**	**95% CI**	***p***	**OR**	**95% CI**	***p***	**OR**	**95% CI**	***p***
Group, IFX	1.17	0.34 to 4.03	0.80	1.22	0.35 to 4.26	0.75	1.02	0.26 to 3.96	0.98	1.16	0.30 to 4.55	0.83
Baseline CES-D, increased^†^	8.18	2.06 to 32.44	< 0.01	9.33	2.09 to 41.77	< 0.01	7.22	1.88 to 27.83	< 0.01	8.73	1.93 to 39.43	< 0.01
Time, 6 weeks	1.09	0.04 to 27.97	0.96	1.35	0.08 to 23.82	0.84	1.11	0.04 to 31.26	0.95	1.34	0.07 to 25.60	0.85
Time, 12 weeks	1.34	0.13 to 14.07	0.81	1.18	0.13 to 11.11	0.88	1.29	0.11 to 15.06	0.84	1.18	0.12 to 11.48	0.88
Time, 24 weeks	4.18	0.26 to 67.75	0.31	4.70	0.29 to 75.59	0.27	3.95	0.24 to 65.05	0.34	4.58	0.28 to 76.18	0.29
Group*time (6 weeks)	0.17	0.00 to 6.66	0.35	0.26	0.01 to 7.66	0.43	0.23	0.01 to 10.97	0.46	0.28	0.01 to 10.01	0.49
Group*time (12 weeks)	0.16	0.01 to 2.89	0.22	0.36	0.02 to 8.55	0.53	0.25	0.01 to 5.92	0.39	0.40	0.01 to 11.01	0.59
Group*time (24 weeks)	0.02	0.00 to 0.72	0.03	0.03	0.00 to 1.32	0.07	0.03	0.00 to 1.30	0.07	0.03	0.00 to 1.80	0.09
BASDAI	−	−	−	1.35	0.94 to 1.93	0.10	−	−	−	1.31	0.89 to 1.95	0.17
CRP, mg/L	−	−	−	−	−	−	1.02	0.99 to 1.05	0.21	1.01	0.97 to 1.04	0.67

*CES-D* Center for Epidemiologic Studies Depression Scale, *CRP* C-reactive protein, *95% CI* 95% confidence interval, *IFX* infliximab, *BASDAI* Bath Ankylosing Spondylitis Disease Activity Index, *OR* odds ratio

*Model B is an extension of model A, with the addition of BASDAI as covariable. Model C is an extension of model A, with the addition of CRP as covariable. Model D is an extension of model A, with the addition of both BASDAI and CRP as covariables

^†^Increased CES-D defined as a score ≥ 16, indicating possible depression

When exploring the odds of possible depression (CES-D score ≥ 16) as the outcome, while adjusting for baseline CES-D status (increased (> 16) vs normal (< 16)), findings were similar: infliximab treatment was associated with a larger reduction in odds of having an increased CES-D after 24 weeks (model 2A: OR_group*time(24)_ = 0.02, 95%CI 0.00 to 0.72, *p* = 0.03), but after adjustment for BASDAI, this association was no longer significant (model 2B: OR_group_ = 0.03, 95%CI 0.00 to 1.32, *p* = 0.07) (Table 2). After adjustment for CRP instead of BASDAI, a similar observation was made (model 2C: OR_group*time(24)_ = 0.03, 95%CI 0.00 to 1.30, *p* = 0.07) (Table 2). Adjustment for both BASDAI and CRP resulted in none of these (treatment or BASDAI or CRP) being significantly associated with increased CES-D scores (model 2D, Table 2).

Analyses using BASFI instead of BASDAI led to similar results: after adjustment for BASFI, the initial association between treatment group and CES-D scores was no longer observed, and when both BASFI and CRP were included, BASFI was associated with CES-D, while CRP was not (models 3A–3D, Table 3). Using the odds of possible depression (CES-D score ≥ 16) as outcome yielded similar results, although BASFI remained associated with possible depression, also after adjustment for CRP (models 4A–4D, Table 3).

**Table 3:** Multivariable GEE analysis exploring the effect of infliximab on CES-D scores, BASFI models

**Outcome = CES-D (absolute score)**		**Model 3A**			Model 3B*			Model 3C*			Model 3D*	
	***B***	**95% CI**	***p***	***B***	**95% CI**	***p***	***B***	**95% CI**	***p***	***B***	** 95% CI**	***p***
Group, IFX	− 0.39	− 2.01 to 1.23	0.63	0.87	− 1.97 to 3.71	0.55	− 0.80	− 2.85 to 1.25	0.44	1.16	− 1.87 to 4.19	0.45
Baseline CES-D, score	0.78	0.46 to 1.11	< 0.01	0.74	0.53 to 0.95	< 0.01	0.77	0.43 to 1.11	< 0.01	0.75	0.53 to 0.96	< 0.01
Time, 6 weeks	− 2.30	− 8.09 to 3.49	0.44	− 1.62	− 6.31 to 3.07	0.50	− 2.31	− 8.14 to 3.53	0.44	− 1.59	− 6.22 to 3.04	0.50
Time, 12 weeks	− 0.42	− 5.63 to 4.78	0.87	0.43	− 4.01 to 4.87	0.85	− 0.58	− 5.88 to 4.72	0.83	0.55	− 3.87 to 4.96	0.81
Time, 24 weeks	0.62	− 4.69 to 5.92	0.82	0.92	− 3.85 to 5.70	0.71	0.42	− 4.92 to 5.75	0.88	1.05	− 3.75 to 5.86	0.67
Group*time (6 weeks)	− 2.94	− 9.23 to 3.35	0.36	− 0.84	− 6.15 to 4.46	0.75	− 2.24	− 8.92 to 4.44	0.51	− 1.20	− 6.53 to 4.14	0.66
Group*time (12 weeks)	− 4.22	− 10.32 to 1.87	0.17	− 2.19	− 7.50 to 3.12	0.42	− 3.27	− 9.69 to 3.16	0.32	− 2.69	− 7.84 to 2.47	0.31
Group*time (24 weeks)	− 6.63	− 13.35 to 0.09	0.05	− 3.89	− 10.04 to 2.27	0.22	− 5.71	− 12.53 to 1.12	0.10	− 4.34	− 10.38 to 1.69	0.16
BASFI	−	−	−	1.56	0.81 to 2.31	< 0.01	−	−	−	1.61	0.80 to 2.42	< 0.01
CRP, mg/L	−	−	−	−	−	−	0.04	− 0.02 to 0.10	0.24	− 0.02	− 0.08 to 0.04	0.46
**Outcome = CES-D (increased vs. normal)**^**†**^		**Model 4A**			Model 4B*			Model 4C*			Model 4D*	
	**OR**	**95% CI**	***p***	**OR**	**95% CI**	***p***	**OR**	**95% CI**	***p***	**OR**	**95% CI**	***p***
Group, IFX	1.17	0.34 to 4.03	0.80	1.59	0.42 to 6.06	0.50	1.02	0.26 to 3.96	0.98	1.60	0.37 to 6.97	0.53
Baseline CES-D, increased^†^	8.18	2.06 to 32.44	< 0.01	12.57	2.11 to 74.95	< 0.01	7.22	1.88 to 27.83	< 0.01	12.68	1.94 to 82.94	< 0.01
Time, 6 weeks	1.09	0.04 to 27.97	0.96	1.43	0.07 to 29.08	0.82	1.11	0.04 to 31.26	0.95	1.43	0.07 to 28.96	0.82
Time, 12 weeks	1.34	0.13 to 14.07	0.81	1.76	0.25 to 12.46	0.57	1.29	0.11 to 15.06	0.84	1.77	0.25 to 12.58	0.57
Time, 24 weeks	4.18	0.26 to 67.75	0.31	4.73	0.38 to 58.18	0.23	3.95	0.24 to 65.05	0.34	4.74	0.37 to 60.52	0.23
Group*time (6 weeks)	0.17	0.00 to 6.66	0.35	0.21	0.01 to 7.10	0.38	0.23	0.01 to 10.97	0.46	0.21	0.01 to 7.91	0.40
Group*time (12 weeks)	0.16	0.01 to 2.89	0.22	0.21	0.01 to 3.18	0.26	0.25	0.01 to 5.92	0.39	0.21	0.01 to 4.14	0.30
Group*time (24 weeks)	0.02	0.00 to 0.72	0.03	0.02	0.00 to 0.79	0.04	0.03	0.00 to 1.30	0.07	0.02	0.00 to 1.13	0.06
BASFI	−	−	−	1.49	1.07 to 2.09	0.02	−	−	−	1.50	1.02 to 2.21	0.04
CRP, mg/L	−	−	−	–	−	−	1.02	0.99 to 1.05	0.21	1.00	0.97 to 1.03	0.96

*CES-D* Center for Epidemiologic Studies Depression Scale, *CRP* C-reactive protein, *95% CI* 95% confidence interval, *IFX* infliximab, *BASFI* Bath Ankylosing Spondylitis Functional Index, *OR* odds ratio

*Model B is an extension of model A, with the addition of BASFI as covariable. Model C is an extension of model A, with the addition of CRP as covariable. Model D is an extension of model A, with the addition of both BASFI and CRP as covariables

^†^Increased CES-D defined as a score ≥ 16, indicating possible depression

## Discussion

This study showed that depressive symptoms are common in patients with AS that have high disease activity. Infliximab improved depressive symptoms in patients with AS after 24 weeks of treatment. This effect could largely be explained by the effect of infliximab on self-reported symptoms of AS.

Over half of the patients with AS and active disease had a CES-D score > 16 at baseline, indicative for possible depression. This high proportion seems to be on the upper end of the prevalence range as reported in the literature, but is likely the result of the inclusion criteria for ASSERT, which required patients to have active disease [1]. For comparison, in control populations in the Netherlands, a possible depression as measured by increased CES-D score has been reported in 5–22% [21–23].

The reduction in depressive symptoms in the infliximab group occurred already within the first weeks and was maintained during the remainder of follow-up. Importantly, treatment with infliximab was not only associated with a decrease in depressive symptoms, but also with decreased odds of CES-D scores above the threshold for (probable) depression. The substantial reduction (an estimated 98% reduction in odds of having an increased CES-D after 24 weeks of weeks of infliximab compared to placebo), although certainly an overestimation of the effect of infliximab on true clinical depression, seems clinically relevant. This implicates that, at least in a population of patients with active AS, infliximab not only decreases the severity of depressive symptoms and odds of probable depression, but potentially even lowers the odds of true clinical depression. Of note, even after up to 54 weeks of treatment, 20–25% of both groups still had CES-D scores suggestive of possible depression.

Comparing our results with other studies that investigated the effect of TNFi on depressive symptoms in AS is difficult, because other study designs and/or instruments were used to assess depressive symptoms (in none the CES-D was used) [6–10]. Two RCTs assessed the effect of etanercept on depressive symptoms as a secondary outcome or in post hoc analysis. The first study, comparing sulfasalazine to etanercept among 566 patients with AS, reported significant improvements in depressive symptoms after 16 weeks in both treatment arms, with larger improvements in the etanercept group [9]. In the second study, where 215 patients with nr-axSpA were treated with etanercept or placebo for 12 weeks followed by etanercept for all subjects for another 12 weeks, depressive symptoms did not differ between groups after 12 weeks or 24 weeks, but had improved from baseline after 24 weeks in both groups [10]. In addition, three observational studies on the effect of infliximab on depressive symptoms in AS were published, with a total of 52 patients and all of limited duration (6–12 weeks) [6–8]. In line with our findings, in these observational studies, similar proportions of patients with depression scores above the threshold for the respective depression instrument used at baseline and over time were observed [7, 8]. Also, improvements in depressive symptoms occurred already within the first weeks after initiation of infliximab. Of note, none of these studies explored whether these improvements can be fully explained by improvements in symptoms or inflammation.

It remains challenging whether our results can help to understand if AS has a direct (inflammatory) or indirect (through pain and limitations) effect on depressive symptoms. The association between treatment with infliximab and improvement of depressive symptoms largely disappeared after adjustment for AS-specific symptoms, i.e. self-reported disease activity and physical function, suggesting the change in depressive symptoms in these patients is (at least partially) secondary to changes in their AS-related symptoms. Of note, the relationship between self-reported depressive symptoms and self-reported AS-related symptoms could (partially) be bidirectional. In rheumatoid arthritis, for example, the patient visual analog scale component of the Disease Activity Score (DAS28) has been strongly associated with depression [24]. Likewise, in the current study, responses on BASDAI or BASFI could be influenced by the patient’s emotional state. Additionally, it is known some covariance exists between all self-reported measures, which likely adds to some overestimation of the observed associations. Notwithstanding, when considering an alternative explanation for the improvements in depressive symptoms, i.e. a reduction in inflammation, CRP did not seem to independently/directly contribute to the effect of infliximab treatment on depressive symptoms: adjustment for CRP did have a minor impact on the association between infliximab and depressive symptoms when compared to the effect of BASDAI or BASFI, suggesting little mediation of the effect of infliximab via CRP-mediated inflammation. This further suggests the mechanism behind depressive symptoms in these patients is mainly based on the impact of AS-related symptoms. It should be noted, however, that due to the small sample size, these secondary analyses were only exploratory and no elaborate path analyses could be conducted. Nonetheless, the association between CRP and depressive symptoms, compared with BASDAI or BASFI, was not only statistically non-significant but also very small numerically, suggesting a direct effect of CRP-mediated inflammation would be little (if any) in a larger sample. Overall, it remains challenging to unravel the intricate relationships between the inflammatory pathophysiological process behind AS, AS-related symptoms, and depression. While CRP is commonly used as inflammatory biomarker in axSpA, we cannot rule out CRP is not appropriate as a biomarker to identify a potential link between inflammation and depression in this disease. While the current results do not allow us to draw firm conclusions regarding these associations between markers of inflammation and depression, our data suggests other inflammatory biomarkers are likely more interesting to further explore than CRP.

In addition, two other interesting observations can be made. First, while all patients had high BASDAI and BASFI scores at baseline, only half of these patients had CES-D scores above the threshold for possible depression, indicating the relation between experienced pain or functional limitations and depression is not an absolute one. Second, baseline CES-D remained strongly associated with the course of CES-D over time, even when accounting for BASDAI or BASFI. This suggests that, while the effect of infliximab on depressive symptoms seems to be mostly mediated by improvement in pain and functional limitations, depressive symptoms as measured with the CES-D are additional and distinct phenomena rather than only a reflection of these pain and limitations. It would be interesting to further explore which patients with active disease have an increased susceptibility to depression, for example as a result of genetic predisposition or personality and coping traits [25]. On this line, it has recently been shown that illness perceptions have an important role in the relationship between back pain and mental health outcomes [26]. Further, in axSpA, patients with comorbid depression are much more likely to suffer from other mental health and substance abuse disorders, also suggesting an underlying vulnerability [27].

The main limitation of this study is the small number of patients restricting the power to detect significant changes and limiting the number of covariables that could be included in the models. However, strong and clinically relevant absolute changes and improvements in depressive symptoms were observed. Further, we should realize that the CES-D is a screening questionnaire which cannot be used to diagnose depression, for which the gold standard still is psychiatric interviewing and examination. On this line it should be noted the questions in the CES-D refer to the past week, which might further reflect reactive depressive symptoms (instead of clinical depression/chronic depression), overestimating the proportion with actual depression. The associations as observed in the analyses of the current study are possibly an overestimation (numerically), and the effect of infliximab on true (physician-diagnosed) clinical depression might be smaller in daily practice. Finally, we did not have data available on the individual BASDAI items, precluding analysis of the contribution of each item.

The findings in the present study have several implications. Rheumatologists should be aware of the high prevalence of depressive symptoms in patients with AS and active disease, while considering that these symptoms are not strictly a result of pain and loss of functioning. In addition, our results suggest that treatment with a TNFi is beneficial for depressive symptoms in the majority of this population. Still, a proportion of patients seem to maintain clinically significant depressive symptoms despite TNFi treatment, which might require specialized treatment.

## Conclusions

The prevalence of depressive symptoms was high among this patient population with active AS. TNFi treatment improved the depressive symptoms of AS patients; our data suggest that this benefit seems an indirect effect of TNFi treatment on AS-related symptoms. Appropriate management of depression in AS deserves attention in clinical practice.

## Supplementary information

**Additional file 1.** CES-D subscale scores over time. Table and graph with CES-D subscale scores over time, by group.

**Additional file 2.** Comparison of ASSERT subjects who did or did not participate in the ancillary depression study. Table with patient characteristics of ASSERT subjects who did or did not participate in the ancillary depression study.

## Data Availability

All data generated or analyzed during this study are included in this published article and its supplementary information files.

## Abbreviations

AS: Ankylosing spondylitis
ASSERT: Ankylosing Spondylitis Study for the Evaluation of Recombinant Infliximab Therapy
BASDAI: Bath Ankylosing Spondylitis Disease Activity Index
BASFI: Bath Ankylosing Spondylitis Functional Index
CES-D: Center for Epidemiologic Studies Depression scale
CRP: C-reactive protein
GEE: Generalized estimating equation
nr-axSpA: Non-radiographic axial spondyloarthritis
RCT: Randomized controlled trial
TNF: Tumor necrosis factor
TNFi: Tumor necrosis factor alpha inhibitor
