# Supplementary material for: Infliximab treatment reduces depressive symptoms in patients with ankylosing spondylitis: an ancillary study to a randomized controlled trial (ASSERT)
Source: Arthritis Res Ther. 2020 Sep 29;22:225. doi: 10.1186/s13075-020-02305-w (PMC7523309; doi:10.1186/s13075-020-02305-w)
Supplement: Supplementary file 1 — Additional file 1. CES-D subscale scores over time. Table and graph with CES-D subscale scores over time, by group. [file 13075_2020_2305_MOESM1_ESM.docx]

**Additional File 1. CES-D subscale scores over time, by group**

**Table. Mean CES-D subscale scores over time, by treatment group**

|  | **Subscale 1 Somatic-retarded activity (0-21)** | | **Subscale 2 Depressed affect (0-15)** | | **Subscale 3 Positive affect (0-12)** | | **Subscale 4 Interpersonal affect (0-6)** | |
| --- | --- | --- | --- | --- | --- | --- | --- | --- |
| **Time** | **IFX** | **PBO** | **IFX** | **PBO** | **IFX** | **PBO** | **IFX** | **PBO** |
| **Week 0** | 7.1 (3.8) | 8.0 (3.9) | 2.4 (2.1) | 2.3 (2.0) | 4.4 (3.0) | 5.6 (2.8) | 0.6 (0.8) | 0.6 (0.5) |
| **Week 6** | 4.6 (3.8)* | 9.4 (3.2)* | 1.7 (2.1) | 2.2 (2.1) | 2.7 (2.6) | 3.3 (2.2) | 0.8 (1.6) | 0.0 (0.0) |
| **Week 12** | 4.5 (4.6)* | 8.8 (3.0)* | 1.9 (2.8) | 2.5 (2.0) | 3.4 (3.0) | 4.0 (2.7) | 0.4 (0.7) | 0.3 (0.5) |
| **Week 24** | 4.9 (4.9)* | 9.9 (4.2)* | 1.5 (2.5) | 2.3 (1.5) | 1.9 (2.6) | 3.7 (2.9) | 0.4 (1.0) | 0.1 (0.4) |
| **Week 54** | 4.5 (4.7) | 4.2 (5.8) | 1.3 (1.6) | 1.0 (2.2) | 2.3 (2.5) | 2.4 (3.9) | 0.3 (0.4) | 1.2 (1.3) |

All data are expressed as mean (SD).
*Significant difference between treatment groups (not adjusted for multiple testing)

**
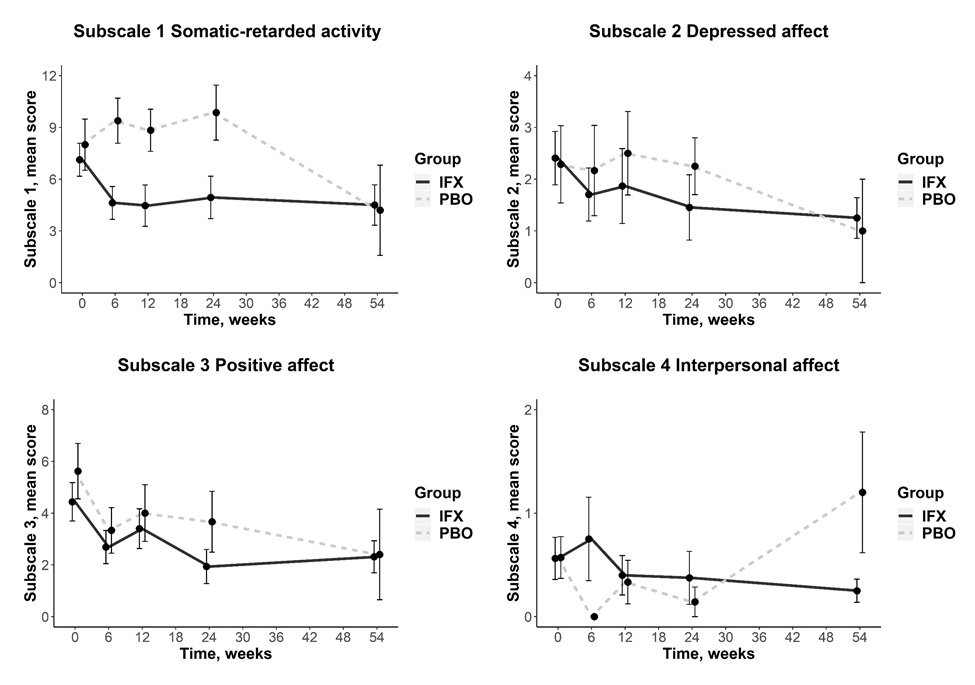
**

**Figure. Mean scores of each CES-D subscale over time, by treatment group**
