# Supplementary material for: Infliximab treatment reduces depressive symptoms in patients with ankylosing spondylitis: an ancillary study to a randomized controlled trial (ASSERT)
Source: Arthritis Res Ther. 2020 Sep 29;22:225. doi: 10.1186/s13075-020-02305-w (PMC7523309; doi:10.1186/s13075-020-02305-w)
Supplement: Supplementary file 2 — Additional file 2. Comparison of ASSERT subjects who did or did not participate in the ancillary depression study. Table with patient characteristics of ASSERT subjects who did or did not participate in the ancillary depression study. [file 13075_2020_2305_MOESM2_ESM.docx]

**Additional File 2. Comparison of ASSERT subjects who did or did not participate in the ancillary depression study**

|  |  | **Ancillary study** | |  |
| --- | --- | --- | --- | --- |
| **Variable*** | **Total**  **(n=279)** | **Yes**  **(n=23)** | **No**  **(n=256)** | **p**† |
| Gender, male | 225 (81%) | 19 (83%) | 206 (80%) | 1.00 |
| Age | 39.8 (10.2) | 40.0 (10.5) | 39.8 (10.2) | 0.93 |
| Disease duration | 10.6 (8.5) | 9.3 (7.9) | 10.8 (8.6) | 0.36 |
| BASDAI | 6.4 (1.6) | 7.0 (1.1) | 6.4 (1.6) | 0.06 |
| BASFI | 5.8 (2.0) | 6.6 (1.5) | 5.7 (2.0) | 0.03 |
| BASMI | 4.1 (2.1) | 4.3 (1.6) | 4.1 (2.1) | 0.47 |
| Patient Global | 6.8 (1.8) | 7.1 (1.4) | 6.7 (1.8) | 0.42 |
| CRP | 24.2 (27.2) | 23.0 (22.1) | 24.3 (27.6) | 0.99 |
| ASAS20 Response, week 24‡ | 121 (61%) | 8 (50%) | 113 (62%) | 0.34 |

All values are expressed as mean (SD) unless otherwise stated.

*Baseline variables, unless otherwise stated.

†For comparison of those who participated in the ancillary depression study vs. those who did not participate.

‡Only in patients randomized to IFX treatment at baseline

ASSERT, Ankylosing Spondylitis Study for the Evaluation of Recombinant Infliximab Therapy; BASDAI, Bath Ankylosing Spondylitis Disease Activity Index; BASFI, Bath Ankylosing Spondylitis Functional Index; BASMI, Bath Ankylosing Spondylitis Metrology Index; CRP, C-reactive protein; ASAS, Assessment of SpondyloArthritis international Society.
